# Synthesis of 2,1-benzisoxazole-3(1*H*)-ones by base-mediated photochemical N–O bond-forming cyclization of 2-azidobenzoic acids

**DOI:** 10.3762/bjoc.12.86

**Published:** 2016-05-04

**Authors:** Daria Yu Dzhons, Andrei V Budruev

**Affiliations:** 1 Chemistry Department, Lobachevsky State University of Nizhny Novgorod, Gagarina pr. 23, Building 5, 603950, Nizhny Novgorod, Russian Federation

**Keywords:** aryl azides, azepinones, 2,1-benzisoxazolones, 1,5-electrocyclization, nitrenes, photochemical cyclization

## Abstract

The base-mediated photochemical cyclization of 2-azidobenzoic acids with the formation of 2,1-benzisoxazole-3(1*H*)-ones is reported. The optimization and scope of this cyclization reaction is discussed. It is shown that an essential step of the ring closure of 2-azidobenzoic acids is the formation and photolysis of 2-azidobenzoate anions.

## Introduction

**Figure 1:**
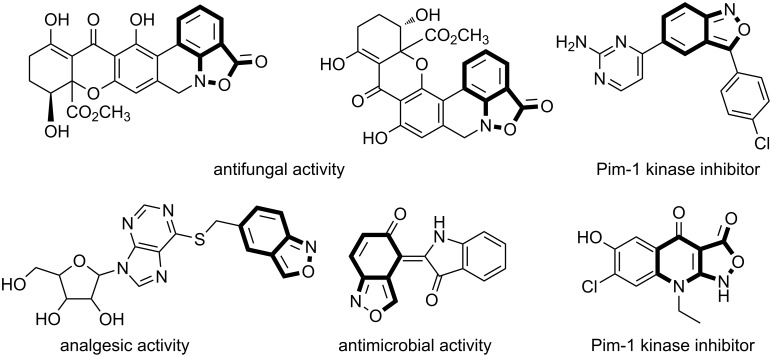
Selected examples of biologically active, fused 2,1-isoxazole derivatives.

Substituted 2,1-benzisoxazoles display diverse biological activity [1–6] (Figure 1) and are widely used as starting materials for the synthesis of important heterocyclic pharmacophores, such as acridines [7–8], quinolines [9–13] and quinazolines [14–16]. Therefore, the search for new methods leading to 2,1-benzisoxazoles is of great interest.

For the preparative synthesis of 2,1-benzisoxazoles, in addition to the traditional method based on the reductive heterocyclization of *ortho*-substituted nitro compounds [17–21], two other routes are available: the annulation of nitroso compounds [22–23] and the thermal [24], catalytic [25–27] or photochemical cyclization of aryl azides [28–31].

However, the presence of electron-withdrawing substituents in the 3-, 5- and 7-position of the benzisoxazole dramatically reduces the thermal stability of these compounds. Indeed, these compounds are the most labile representatives of several isomeric oxazoles [32], and they begin to decompose at temperatures slightly above 30 °C, which limits the number of methods for their preparation. Thus the search for new methods for the synthesis of substituted 2,1-benzisoxazoles under mild reaction conditions is required.

**Scheme 1:**
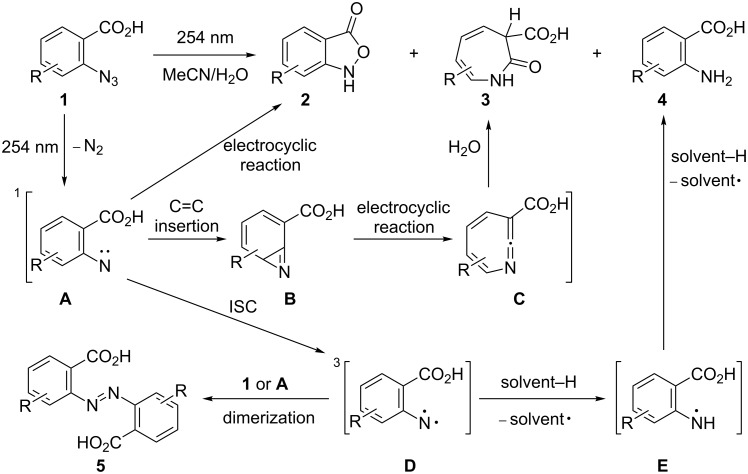
Photochemical cyclization of substituted 2-azidobenzoic acids and possible reaction mechanism [34–59].

Previously, in the investigation of the photochemical cyclization of 2-azidobenzoic acid (**1a**) using aqueous organic solvent mixtures (Scheme 1), the formation of the cyclization products such as 2,1-benzisoxazole-3(1*H*)-one (**2a**) and 2-oxo-3-carboxy-3*H*-azepine (**3a**) has been reported [30]. The structure of benzisoxazole **2a** was determined by IR, ^1^H and ^13^C NMR spectroscopy and by comparison of its mass spectrum with the corresponding spectrum from the NIST library (NIST: 37717). In addition, the structure was confirmed by the alternative synthesis of **2a** through the heterocyclization of 2-nitrobenzoic acid [28–29] and X-ray structure analysis [33].

The authors observed that the yields of both **2a** and **3a** increased with increasing amount of water as nucleophilic solvent in the reaction mixture [30] and obtained a maximum yield of 20% and 50%, respectively, at a water content of 50%. Under these conditions, no formation of the primary amine **4** (a product of the typical triplet nitrene reaction (Scheme 1, intermediate **D**)) was detected. The replacement of the aprotic solvent dioxane with acetonitrile or THF did not affect the yield of the cyclization products. The photolysis of **1a** (Scheme 1, intermediate **A**) resulted in low yields of **2a** because of the competitive formation of reaction products.

In the past, research has been focused on the formation of 2-substituted 3*H*-azepines **3** as products of the photolysis or thermolysis of aromatic azides [34–35]. The proposed mechanism for their formation was confirmed by identification of the reaction intermediates using low-temperature and time-resolved spectroscopy [36–43]. It is currently believed that azepines are formed through the singlet nitrogen pathway of the reaction (Scheme 1, path: **1** → **A** → **B** → **C** → **3**) [44–49].

These nitrenes form benzazirines [19] (Scheme 1, intermediate **B**) that rearrange into cyclic ketenimines – 1,2-didehydroazepines (Scheme 1, intermediate **C**) [50]. According to quantum-chemical calculations, the energy barrier of this rearrangement is approximately 40 kJ mol^−1^ [51], with the limiting step being the formation of **B**. Therefore, the direction of this reaction solely depends on the conversion of **A** and formation of **C**.

Dyall et al. [52–53] have proposed a pericyclic mechanism for the formation of heterocyclic compounds in the pyrolysis of aryl azides with unsaturated *ortho*-substituents. A possible reaction mechanism for the photochemical formation of **2** (Scheme 1, path: **1** → **A** → **2**) based on the report by Platz et al. [54] includes the benzofuroxan formation by photolysis of 2-azidonitrobenzene through the intermediate singlet nitrene **A** (Scheme 1) without the formation of other intermediates.

The formation of substituted 2,1-benzisoxazoles from aryl azides was reported for the first time by Smith et al. [24] in the synthesis of 3-phenyl-2,1-benzisoxazole (3-phenylanthranil) by thermolysis of 2-azidobenzophenone. In another work [55], the photochemical formation of 3-amino-6-nitro-2,1-benzisoxazole starting from 2-azido-4-nitrobenzamide was observed. The authors subsequently investigated the multiplicity of the involved nitrene by repeating the reaction in the presence of isoprene as a triplet nitrene quencher. The addition of isoprene lead to a significantly increased yield of 3-amino-6-nitro-2,1-benzisoxazole and an insignificant decrease of the primary amine yield. Thus it was demonstrated that the formation of 3-amino-6-nitro-2,1-benzisoxazole goes through an intermediate singlet nitrene.

Possibly, similar to the benzofuroxan and 3-amino-6-nitro-2,1-benzisoxazole formation, the carboxylate group of **A** (Lewis base) donates an electron lone pair to the electron-deficient singlet nitrene fragment of **A** (Lewis acid) with formation of the N–O bond in **2** through a 1,5-electrocyclization reaction [56–57].

Nonreacted singlet nitrenes **A** may undergo intersystem crossing (ISC) into the less reactive triplet state (Scheme 1, intermediate **D**). Although a multiplicity change is a spin-forbidden transition, it can be partially allowed in some cases. According to another report [39], the major products formed from triplet nitrenes are primary amines **4** through hydrogen-atom abstraction [58], secondary amines, 1,2-arylhydrazides and azo compounds **5**, which are obtained by recombination of radicals [59]. Moreover, it was shown that yields of primary amines increased in the photolysis reactions of aryl azides without participating *ortho*-substituents in hydrocarbons as the solvents [39].

In the present research it is demonstrated that using ethanol as the solvent for the photolysis reaction leads to benzisoxazole **2a** with an increased yield of 35% and the yield can be further improved to 40% by the addition of a base. Thus, the optimization of the reaction conditions of the base-mediated photochemical synthesis of substituted 2,1-benzisoxazole-3(1*H*)-ones **2** has been performed.

## Results and Discussion

For optimizing the reaction conditions for the synthesis of **2a** by photolysis of **1a**, the reaction was performed in different solvents in the absence or presence of a base. As solvents, alcohols and aqueous organic solvent mixtures were tested and alkali metal hydroxides, carbonates or acetates were screened as the base. All reactions were carried out by irradiating the base suspended in the solution of **1a** with a mercury low-pressure quartz lamp (254 nm) in a quartz reactor with intensive stirring.

It was found that the yields of **2a** after photolysis of **1a** substantially increased in the presence of a base. No dependency on the nature of the base could be observed and the yield did not improve further when more than 1 equivalent of the base was used (Table 1). Without irradiation, the reaction did not proceed at all. Replacing EtOH as the solvent with iPrOH did not change the yield of **2a**. A chromatographic separation of the reaction mixture obtained by photolysis of **1a** in alcohols showed that **2a** had formed as the sole product. In this case, neither the formation of **3a** nor the corresponding 2-ethoxy- or 2-isopropoxy-substituted azepines could be detected.

**Table 1:** Optimization of conditions for the synthesis of substituted 2,1-benzisoxazole-3(1*H*)-ones.^a^

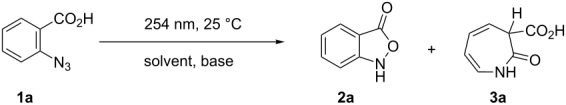					

Entry	Base (equiv)	Solvent	Yield (**2a**)^b^	Yield (**3a**)^b^	*t*, min^c^

1	–	1,4-dioxane	–	–	60
2	–	EtOH	38%	1%	60
3	K_2_CO_3_ (0.5)	EtOH	63%	–	60
4	K_2_CO_3_ (1)	EtOH	75%	–	60
5	K_2_CO_3_ (1)	iPrOH	75%	–	60
6	K_2_CO_3_ (5)	1,4-dioxane/water 1:1 (v/v)	43%	8%	60
7	K_2_CO_3_ (3)	EtOH	75%	–	60
8	Na_2_CO_3_ (0.5)	EtOH	65%	–	60
9	Na_2_CO_3_ (1)	EtOH	73%	–	60
10	NaHCO_3_ (0.5)	EtOH	70%	–	60
11	NaHCO_3_ (1)	EtOH	70%	–	60
12	KOH (1)	EtOH	60%	–	60
13	NaOH (1)	Water	30%	12%	60
**14**	**NaOAc (1)**	**EtOH**	**75%**	–	60
15	NaOAc (1)	1,4-dioxane/water 1:1 (v/v)	63%	20%	60

^a^Reaction conditions: **1a** (0.78 mmol), solvent (15.0 mL); UV light (2 × 15 W Hg low-pressure lamp (254 nm), UV intensity was approximately 7 mW/cm^2^). ^b^Yields were determined by HPLC analysis using an external standard. ^c^Irradiation time*:* the degree of conversion of **1a** was 100%.

The photolysis in a mixture of 1,4-dioxane/water led to a decreased yield of **2a** with a simultaneous increase in the yield of **3a**. In our opinion, this effect of the solvent or base on the yield of **2a** may be explained by the formation of **2a** through a heterocyclization of 2-azidobenzoate anions. Therefore, the role of the base in the reaction is the in situ generation of the 2-azidobenzoate anion and the solvent efficiently supports this formation.

The best yield of **2a** was obtained by photolysis of **1a** in ethanol in the presence of sodium acetate (Table 1, entry 14).

With the optimal conditions at hand, we next investigated the scope of the cyclization using differently substituted 2-azidobenzoic acids (Table 2). The desired products **2b**–**f** were isolated in moderate to high yields (Table 2). Both electron-withdrawing groups (Cl, Br, I) and the electron-donating group (triphenylmethyl) were well-tolerated.

**Table 2:** Substrate scope for the heterocyclization of 2-azidobenzoic acid.^a^

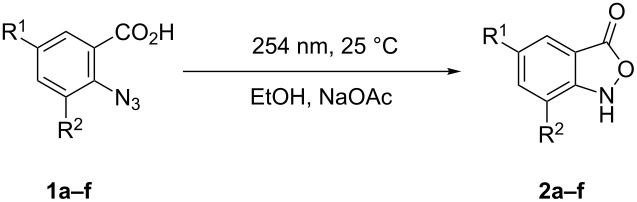				

Entry	R^1^	R^2^	Yield (**2**)^b^	*t*, min^c^

1 (**1a**)	H	H	75% (**2a**)	60
2 (**1b**)	Cl	Cl	92% (**2b**)	60
3 (**1c**)	Br	H	68% (**2c**)	60
4 (**1d**)	Br	Br	62% (**2d**)	60
5 (**1e**)^d^	I	H	51% (**2e**)	60
6 (**1f**)	Tr^e^	H	39% (**2f**)	60

^a^Reaction conditions: **1** (0.78 mmol), NaOAc (1 equiv), EtOH 96% (15.0 mL); UV light (2 × 15 W Hg low-pressure lamp (254 nm), UV intensity was approximately 7 mW/cm^2^). ^b^Yield of the isolated product. ^c^Irradiation time*:* the degree of conversion of **1a**–**f** was 100%. ^d^For the optimal conditions for synthesis of **2e** see Table 4, entry 10. ^e^Tr – triphenylmethyl group (trityl group).

The thermal stability of the products of **2** decreases in the series: **2f** > **2a** > **2e** > **2d** > **2c** > **2b**. Halogen-substituted compounds **2b**–**e** decompose at room temperature within about 5–30 min and the products **2a**,**f** are stable for a couple of hours.

In general, azides with electron-withdrawing groups tend to give higher yields than those substituted with electron-donating groups (**2b**−**e** vs **2f**, Table 2, entries 2–5 vs entry 6). However, the yield of **2** decreased in the series Cl → Br → I (**2b**–**e**, Table 2, entries 2–5), which was probably a manifestation of the internal photochemical heavy-atom effect [60]. The presence of bromo- or iodo-substituents in the substrates **1c**–**e** increases the probability of intersystem crossing for the initially formed singlet nitrenes (Scheme 1, intermediate **A**) to the triplet state (Scheme 1, intermediate **D**), thus resulting in decreased yields of the cyclization products **2c**–**e**. In these cases, the formation of the triplet nitrene **D** should lead to increased yields of primary amines. However, as can be seen from Table 1, the formation of the latter was not observed when ethanol was used as the solvent. To further test the solvent effect on the outcome of the reaction, we repeated the photolysis of azides **1a**–**f** under the optimized conditions but in aqueous dioxane instead of ethanol. As can be seen in Table 3, the photolysis of **1c**,**d** in a 1,4-dioxane/water mixture under these conditions lead to decreased yields of **2c**,**d** and to the formation of azepine **3c** and primary amines **4c**,**d** with significant yields. An explanation for the decreased yields observed for isoxazoles **2c**,**d** may be the photo-induced breaking of the C–Br bond that completely changed the way of the reaction. However, this possibility does not explain the formation of primary amines **4c**,**d**.

**Table 3:** Scope for photo-induced heterocyclization of 2-azidobenzoic acids in 1,4-dioxane/water mixture in the presence of potassium carbonate.^a^

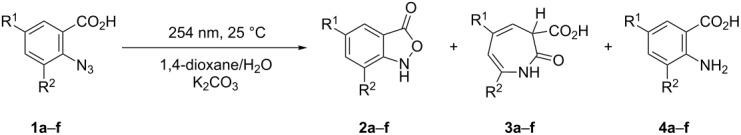						

Entry	R^1^	R^2^	Yield (**2**)^b^	Yield (**3**)^b^	Yield (**4**)^b^	*t*, min^c^

1 (**1a**)	H	H	25% (**2a**)	20% (**3a**)	– (**4a**)	90
2 (**1b**)	Cl	Cl	92% (**2b**)	– (**3b**)	– (**4b**)	60
3 (**1c**)	Br	H	34% (**2c**)	18% (**3c**)	10% (**4c**)	180
4 (**1d**)	Br	Br	62% (**2d**)	– (**3d**)	4% (**4d**)	60
5 (**1e**)	I	H	5% (**2e**)	– (**3e**)	– (**4e**)	120
6 (**1f**)	Tr	H	40 % (**2f**)	– (**3f**)	– (**4f**)	60

^a^Reaction conditions: **1** (0.78 mmol), K_2_CO_3_ (1 equiv), 1,4-dioxane/water 1:1 (v/v) (15.0 mL); UV light (2 × 15 W Hg low-pressure lamp (254 nm), UV intensity was approximately 7 mW/cm^2^). ^b^Yields determined by HPLC analysis using an external standard. ^c^Irradiation time*:* the degree of conversion of **1a**–**f** was 100%.

It should be noted that the photolysis of double *ortho*-substituted azides (Table 3, entries 2, 4, R^2^ ≠ H) did not lead to the formation of azepines **3**. This confirms the suggestions of previous reports [61–62] about the impossibility of ring expansion of such aryl azides.

Interestingly, under these conditions (Table 3), the yields for 3*H*-azepine **3a** and **3c** were determined as 20 and 18%, respectively. We wondered if the yields of these compounds could be improved to allow a preparative synthesis of **3a** and **3c**. For this reason, the reaction conditions were optimized towards azepines **3a** and **3c**. According to previously published reports [30,63–64], the yields of the corresponding 3*Н*-azepines could be increased by increasing the amount of the nucleophile (water) present in the reaction mixture. Thus, we attempted the preparative synthesis of **3a** based on our technique described earlier [30]. After irradiating a solution of azide **1a** in acetonitrile/water (3:7, v/v) for 24 h in a quartz reactor, a complete conversion of starting compound **1a** according to HPLC monitoring was observed. Following work-up and preparative column chromatography azepine **3a** could be isolated in 50% yield. For the preparative synthesis of **3c** the method had to be slightly modified: In this case the reaction was performed in 1,4-dioxane/water (1:10, v/v) solution and irradiated for 1.5 h in a quartz reactor. After this, the conversion of **1c** (according to HPLC monitoring) was found to be 100% and azepine **3c** was isolated after preparative column chromatography in 50% yield (see Supporting Information File 1).

As is also shown in Table 3, the high photochemical sensitivity of both the C–I bond and the azide group present in **1e**, unlike the others, complicates the synthesis of benzisoxazolone **2e**. The photolysis of **1e** under these conditions resulted in the formation of several products in low yields. Therefore, the synthesis towards benzisoxazolone **2e** was reoptimized (Table 4). It was found that increasing the amount and strength of the base resulted in an increased selectivity and reaction rate. Indeed, using 10 equiv of sodium hydroxide as the base in the reaction resulted in a 51% yield of benzisoxazole **2e** at complete photolysis of **1e** (Table 4, entry 10). Under these conditions, in addition to compound **2e**, the formation of **2a** together with some other unidentified products was observed (albeit in low yields). A similar observation has been previously reported by Platz et al. [65].

**Table 4:** Optimization of conditions for the synthesis of 5-iodo-2,1-benzisoxazole-3(1*H*)-one (**2e**).^a^

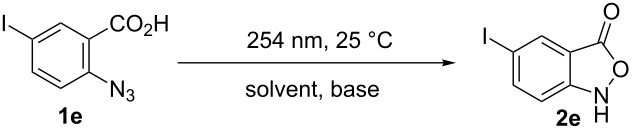				

Entry	Base (equiv)	Solvent	Yield (**2e**)^b^	*t*, min^c^

1	–	1,4-dioxane/water 1:1 (v/v)	6%	60
2	–	EtOH	12%	140
3	NaOAc (1)	EtOH	16%	60
4	NaOAc (1)	EtOH	10%	160
5	NaOAc (1)	1,4-dioxane/water 1:1 (v/v)	5%	60
6	NaOAc (1.4)	EtOH	14%	60
7	NaOAc (1.2)	EtOH	13%	90
8	KOH (2.8)	EtOH	30%	90
9	KOH (2.8)	EtOH	18%	110
**10**	**KOH (10)**	**EtOH**	**51%**	**60**
11	KOH (10)	EtOH	34%	90

^a^Reaction conditions: **1e** (0.78 mmol), solvent (15.0 mL); UV light (2 ×15 W Hg low-pressure lamp (254 nm), UV intensity was approximately 7 mW/cm^2^). ^b^Yields determined by HPLC analysis using an external standard. ^c^Irradiation time*:* the degree of conversion of **1e** was 100%.

**Scheme 2:**
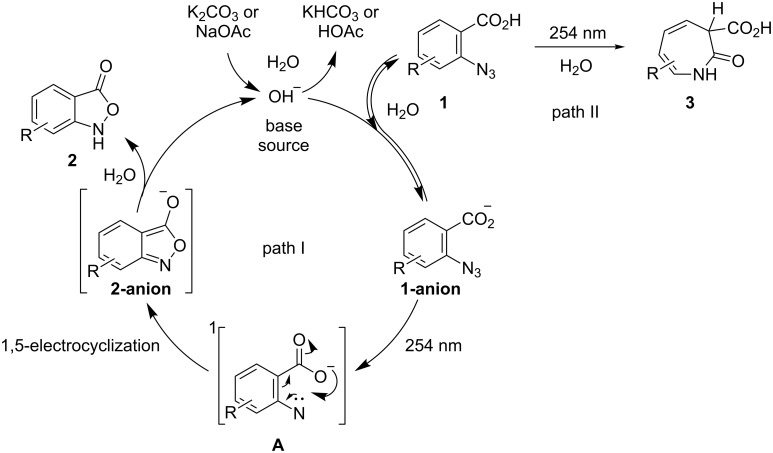
Proposed reaction mechanism of the base-mediated photochemical cyclization of 2-azidobenzoic acids.

Based on the results mentioned above and described in the related reports (Scheme 1), a possible reaction mechanism for the formation of **2** was proposed (Scheme 2). At first, **1** produced a salt of 2-azidobenzoic acid **1** (Scheme 2, **1-anion**) by neutralization of a base. Next, the salt was decomposed by irradiation and the singlet nitrene **A** (Scheme 2, intermediate **A**) was formed. Finally, the electron pair of the carboxylic group (Scheme 2, intermediate **A**) was joined by 1,5-electrocyclization to the electron-deficient singlet nitrene **A** with formation of **2-anion** (see Scheme 2), which was neutralized by water (path I). Thus, the first path of cyclization of **1** was realized.

Meanwhile a molecular form of **1** produced azepine **3** (path II) by irradiation. The detailed mechanism of the formation of **3** is shown in Scheme 2.

Thus, in the photochemical reaction both ionic and molecular forms of **1** can be used. To increase the yield of **2**, it is necessary to shift the equilibrium towards the ionic form **1** (in situ salt formation).

## Conclusion

In summary, we have developed an effective photochemical cyclization strategy for the synthesis of functionalized 2,1-benzisoxazole-3(1*H*)-ones. The present work offers a method to access 2,1-benzisoxazole-3(1*H*)-ones in good yields by using mild reaction conditions at room temperature. The proposed photochemical strategy permits the synthesis the high thermo-labile compounds from the class of 2,1-benzisoxazole-3(1*H*)-ones. Based on the results of the control experiments, it was found that an important stage of the ring closure is the formation of 2-azidobenzoate anion photolysis that results in the heterocyclization product.

## Supporting Information

File 1 Experimental procedures, characterization and spectral data for synthesized compounds.
