# Supplementary material for: Synthesis of 2,1-benzisoxazole-3(1H)-ones by base-mediated photochemical N–O bond-forming cyclization of 2-azidobenzoic acids
Source: Beilstein J Org Chem. 2016 May 4;12:874–81. doi: 10.3762/bjoc.12.86 (PMC4902054; doi:10.3762/bjoc.12.86)
Supplement: File 1 — Experimental procedures, characterization and spectral data for synthesized compounds. [file Beilstein_J_Org_Chem-12-874-s001.pdf]

Supporting Information

for

Synthesis of 2,1-benzisoxazole-3(1*H*)-ones by base-mediated photochemical N–O bond-forming cyclization of 2-azidobenzoic acids

Daria Yu. Dzhons and Andrei V. Budruev*

Address: Chemistry Department, Lobachevsky State University of Nizhny Novgorod, Gagarina pr. 23, build. 5, 603950, Nizhny Novgorod, Russian Federation

Email: Andrei V. Budruev* - budruev@gmail.com

*Corresponding author

Experimental procedures, characterization and spectral data for synthesized compounds

Materials and equipment

Solvents were purified by methods described in [1]. UV–vis spectra were recorded on an Analytik Jena Specord 40 spectrophotometer. Mass spectra were recorded on a Thermo Electron Trace GC Ultra/DSQ II (GC–MS). ^{1}H and ^{13}C NMR spectra were recorded on a Varian 400 MR NMR spectrometer. Chemical shifts (δ) are reported in ppm relative to the residual solvent peak ($CDCl_3$: 7.26 ppm for ^{1}H and 77.16 ppm for ^{13}C; DMSO-d_6: 2.50 ppm for ^{1}H and 29.84 ppm for ^{13}C).

Procedure for HPLC analysis of the reaction mixtures

To 5 mL ethanol was added 1 mL of the reaction mixture and the obtained solution was analyzed by reversed-phase HPLC (Shimadzu LC-20AD equipped with detector (SPD-M20A), loop injection volume of 20 µL and a discovery C-18 column (5 µm, d = 3 mm, l = 25 cm)). A gradient solvent mixture (from EtOH (34.5%)/0.01% aqueous phosphoric (64.5%) to 100% EtOH within 20 min, followed by pure EtOH for 50 min) at a flow rate of 0.15 mL/min was used as the eluent.

An external standard method for the determination of HPLC yields of benzisoxazole, primary amine and azepine formed in the photolysis reaction was used. A calibration curve of different concentrations of the test compound was recorded and used to determine its concentration in the analyzed sample. The determination of the optimal reaction conditions of benzisoxazole and 3*H*-azepine was based on the determination of their HPLC yields. The progress of the photolysis reactions was monitored by analysis of the HPLC peak areas of the starting azides. Isolated yields refer to the yield of benzisoxazole and 3*H*-azepine that were obtained after synthesis under optimized conditions followed by column chromatography.

Synthesis of 2-amino-3,5-dichlorobenzoic acid (4b, CAS: 2789-92-6): the compound was obtained by chlorination of 2-aminobenzoic acid in HCl according to the procedure described in [2]. IR (suspension in nujol, cm^{-1}): 3492, 3372, 1682, 1615, 1575, 1544, 1421, 1402, 1312, 1251, 1227, 1153, 1073, 876, 789.

Synthesis of 2-amino-5-bromobenzoic acid (4c, CAS: 5794-88-7) and **2-amino-3,5-dibromobenzoic acid (4d, CAS: 609-85-8):** A modified synthesis [3] was used. A solution of bromine (7.2 g, 2 mL, 40 mmol) in 47 mL glacial acetic acid was added dropwise to sodium 2-aminobenzoate (6.4 g, 40 mmol) in 32 mL of glacial acetic acid at 15 °C and the mixture was stirred for 1 h at the same temperature. The product was filtered off, washed with benzene and dried in the dark. The bromobenzoic acids containing mixture (0.5 g) was added to 10 mL of boiling water followed by the addition of 1.3 mL of concentrated hydrochloric acid and hot filtration under vacuum. The insoluble material contained 2-amino-3,5-dibromobenzoic acid, whereas 2-amino-5-bromobenzoic acid precipitated upon cooling of the filtrate.

2-Amino-5-bromobenzoic acid (4c) IR (suspension in nujol, cm^{-1}): 3497, 3383, 1675, 1616, 1587, 1548, 1423, 1377, 1316, 1292, 1239, 1160, 1127, 812, 748, 691.

2-Amino-3,5-dibromobenzoic acid (4d) IR (suspension in nujol, cm^{-1}): 3469, 3364, 1682, 1600, 1564, 1538, 1454, 1421, 1378, 1328, 1307, 1226, 1063, 902, 882, 790, 711, 691, 591, 543.

Synthesis of 2-amino-5-iodobenzoic acid (4e, CAS: 5326-47-6): A modified synthesis [4] was used. To the mixture of 2.89 g (18.2 mmol) sodium 2-aminobenzoate, 40 mL acetic acid and 2.31 g (9.1 mmol) molecular iodine, 5 mL (44.1 mmol, 30 wt %) aqueous hydrogen peroxide was added drop wise. The

reaction mixture was stirred at 50 °C for 3 h and poured into 400 mL water. The formed crystals were filtered and recrystallized from benzene to give 2-azido-5-iodobenzoic acid (**2**). IR (suspension in nujol, cm^{-1}): 3500, 3387, 1670, 1612, 1580, 1541, 1419, 1377, 1319, 1290, 1163, 1125, 813, 688, 619.

Synthesis of 2-amino-5-(triphenylmethyl)benzoic acid (4f, CAS: 114202-46-9): Synthesis [5] was used. IR (suspension in nujol, cm^{-1}): 3505, 3386, 3054, 1667, 1590, 1557, 1464, 1445, 1377, 1308, 1245, 1172, 1036, 828, 765, 751, 634, 610.

General procedure 1 for the synthesis of substituted 2-azidobenzoic acids 1a–f: The solution of the corresponding 2-aminobenzoic acid (15.3 mmol) in water (15 mL) and conc. HCl (17 mL) was placed in a 200 mL beaker and cooled to 5 °C. To this mixture a precooled solution of sodium nitrite (1.06 g, 15.3 mmol in 10 mL water) was added drop wise with vigorous stirring. After stirring for 15 min, a solution of sodium azide (1.08 g, 16.6 mmol) in 10 mL water was added and the resulting mixture was gradually warmed to room temperature and stirred at this temperature for about 2 h. The white crystalline azides precipitated from the reaction mixture and were filtered off. After washing with cold water and drying in the dark at room temperature the azides were recrystallized from heptane/benzene (v/v, 1:1) [6].

2-Azidobenzoic acid (1a, CAS: 31162-13-7): Colorless needles. Yield: 78%. The product has m.p. of 144 °C (with decomposition). ^{1}H NMR (400 MHz, $CDCl_3$) δ 10.65 (s, 1H), 8.11 (dd, *J* = 7.9, 1.5 Hz, 1H), 7.66 – 7.56 (m, 1H), 7.31 – 7.21 (m, 2H). ^{13}C NMR (101 MHz, $CDCl_3$) δ 168.8, 140.4, 134.6, 133.4, 125.1, 120.9, 119.7; IR (suspension in nujol oil, cm^{-1}): 2131, 2110, 2081 (ν_{as} N_3); 1692 (ν C=O); 1267 (ν_s N_3).

2-Azido-3,5-dichlorobenzoic acid (1b, CAS: 88279-10-1): Light yellow needles. Yield: 78%. ^{1}H NMR (400 MHz, $CDCl_3$) δ 7.96 (d, *J* = 2.5 Hz, 1H), 7.63 (d, *J* = 2.5 Hz, 1H). ^{13}C NMR (101 MHz, $CDCl_3$) δ 166.5, 136.1, 134.8, 131.6, 131.0, 125.6, 120.9; IR (suspension in nujol oil, cm^{-1}): 2147, 2119, 2075 (v_{as} N_3); 1694 (v C=O); 1292 (v_s N_3).

2-Azido-5-bromobenzoic acid (1c, CAS: 112253-72-2): Pale yellow needles. Yield: 78%. ^{1}H NMR (400 MHz, $CDCl_3$) δ 8.21 (d, *J* = 2.4 Hz, 1H), 7.70 (dd, *J* = 8.6, 2.4 Hz, 1H), 7.16 (d, *J* = 8.6 Hz, 1H). ^{13}C NMR (101 MHz, $CDCl_3$) δ 167.2, 137.3, 136.0, 122.3, 121.4, 117.9; IR (suspension in nujol oil, cm^{-1}): 2144, 2119, 2085 (v_{as} N_3); 1697 (v C=O); 1301 (v_s N_3).

2-Azido-3,5-dibromobenzoic acid (1d, CAS: 830340-88-0): Light yellow needles. Yield: 78%. ^{1}H NMR (400 MHz, $CDCl_3$) δ 8.14 (d, *J* = 2.3 Hz, 1H), 7.95 (d, *J* = 2.3 Hz, 1H). ^{13}C NMR (101 MHz, $CDCl_3$) δ 166.8, 140.5, 138.4, 134.5, 125.9, 120.6, 119.0; IR (suspension in nujol oil, cm^{-1}): 2151, 2112 (v_{as} N_3); 1694 (v C=O); 1296 (v_s N_3).

2-Azido-5-iodobenzoic acid (1e, CAS: 112253-72-2): Yellow plates. Yield: 78%. ^{1}H NMR (400 MHz, $CDCl_3$) δ 10.23 (s, 1H), 8.36 (d, *J* = 1.9 Hz, 1H), 7.87 (dd, *J* = 8.5, 2.0 Hz, 1H), 7.02 (d, *J* = 8.5 Hz, 1H). ^{13}C NMR (101 MHz, $CDCl_3$) δ 167.7, 143.1, 141.8, 140.4, 122.6, 121.6, 87.8, 77.1. IR (suspension in nujol oil, cm^{-1}): 2120, 2081 (v_{as} N_3); 1696 (v C=O); 1308 (v_s N_3)

2-Azido-5-(triphenylmethyl)benzoic acid (1f, new compound): White powder. Yield: 78%. Synthesis according to standard procedure for the 2-azidobenzoic acids using corresponding amine dissolved in 40 mL HCl. ^{1}H NMR (400 MHz, $CDCl_3$) δ

8.05 (d, *J* = 1.8 Hz, 1H), 7.46 (dd, *J* = 8.6, 1.8 Hz, 1H), 7.31 – 7.12 (m, 16H). ^{13}C NMR (101 MHz, $CDCl_3$) δ 167.7, 145.9, 144.4, 137.7, 137.4, 135.2, 132.5, 131.1, 130.9, 129.7, 129.1, 128.5, 127.9, 127.6, 126.4, 126.1, 125.3, 119.7, 118.7, 118.1, 77.4, 77.3, 77.1, 76.8, 64.5, 21.5; IR (suspension in nujol oil, cm^{-1}): 2112, 2081 (ν_{as} N_3); 1691 (ν C=O); 1251 (ν_s N_3).

General procedure 2 for the photochemical experiments

The stirred solution of the azide (ca. 5 mmol/L) in a quartz reactor (diameter 2 cm) was irradiated at room temperature with intensive stirring. As UV light source a 2 × 15 W Hg low-pressure lamp (λ = 254 nm, intensity ca. 7 mW/cm²) was used with a distance of 15 cm. The photolysis reaction was continued until complete decomposition of the starting azide was observed (HPLC monitoring).

Synthesis of 2,1-benzisoxazole-3(1*H*)-one (2a, CAS: 31499-90-8): According to General procedure 2 the mixture of 13.1 mg (0.078 mmol) of 2-azidobenzoic acid and 10.8 mg (0.078 mmol) of potassium carbonate in 10 mL ethanol was irradiated for 1 h with intensive stirring. The reaction mixture was added to 50 mL water, extracted with benzene and eluted through silica gel. The benzisoxazole-containing solution was evaporated in vacuo affording 2,1-benzisoxazole-3(1*H*)-one with 75% yield. Pale yellow crystals reddening at room temperature and stored at −20 °C. ^{1}H NMR (400 MHz, $CDCl_3$) δ 8.69 (s, 1H), 7.85 (d, *J* = 7.8 Hz, 1H), 7.68 (t, *J* = 7.7 Hz, 1H), 7.31 (s, 1H), 7.23 (d, *J* = 8.2 Hz, 1H). ^{13}C NMR (101 MHz, $CDCl_3$) δ 169.0, 155.6, 135.2, 125.8, 124.7, 112.6, 111.9; IR (suspension in nujol oil, cm^{-1}): 3127 (ν NH); 1744, 1719 (ν C=O); 1068 (ν CO); m/z (rel, intensity) 136.02(M^++2, 5.64), 134.91(M^+, 100.00), 118.93(2.73), 103.94(3.61), 90.92(M^+-CO_2, 14.07), 78.94(7.04), 76.97(5.45),

75.94(8.75), 63.98(10.01), 62.96(6.98), 51.99(11.24), 50.97(3.55), 49.95(4.86), 43.99(8.23), 39.95(2.83).

Synthesis of 5,7-dichloro-2,1-benzisoxazole-3(1*H*)-one (2b, new compound): According to General procedure 2 the mixture of 20.1 mg (0.08 mmol) of 2-azido-3,5-dibromobenzoic acid and 11.9 mg (0.08 mmol) of potassium carbonate in 10 mL of ethanol was irradiated for 1 h. The reaction mixture was eluted through silica gel using ethanol and the benzisoxazole-containing solution was evaporated to dryness in vacuo affording 5,7-dichloro-2,1-benzisoxazole-3(1*H*)-one with 92% yield as yellow, thermally unstable crystals. m/z (rel, intensity) 204.92(M^++2, 26.01), 202.91(M^+, 95.23), 160.92(29.51), 158.93(M^+-CO_2, 100.00), 158.00(15.08), 156.93(64.54), 97.22(15.29), 88.12(16.83), 61.15(14.95).

Synthesis of 5-bromo-2,1-benzisoxazole-3(1*H*)-one (2c, new compound): According to General procedure 2 the mixture of 19.4 mg (0.08 mmol) of 2-azido-5-bromobenzoic acid and 11.04 mg (0.08 mmol) of potassium carbonate in 10 mL of ethanol was irradiated for 1 h. The reaction mixture was evaporated to dryness, the residue dissolved in dichloromethane and eluted through silica gel. The benzisoxazole-containing solution was evaporated to dryness in vacuo at 37 °C affording 5-bromo-2,1-benzisoxazole-3(1*H*)-one with 68% yield as yellow crystals. m/z (rel, intensity) 215.01(M^++2, 91.43), 214.11(25.41), 213.01(M^+, 100.00), 212.18(15.07), 171.01(M^++2-CO_2, 74.46), 170.10(16.98), 169.04(M^+-CO_2, 75.75), 168.19(14.10), 149.10(62.84), 144.05(13.76), 142.08(14.72), 90.13(52.42), 78.15(14.82), 63.13(30.95), 62.15(13.37).

Synthesis of 5,7-dibromo-2,1-benzisoxazole-3(1*H*)-one (2d, new compound): According to General procedure 2 the mixture of 17.1 mg (0.053 mmol) of 2-azido-3,5-dibromobenzoic acid and 10 mg (0.072 mmol) of potassium carbonate in 10 mL of ethanol was irradiated for 1 h. The reaction mixture was eluted through silica gel using ethanol. The benzisoxazole-containing solution was evaporated to dryness in vacuo affording 5,7-dibromo-2,1-benzisoxazole-3(1*H*)-one with 62% yield as yellow crystals. m/z (rel, intensity) 294.92(M^++2, 26.01), 292.91(M^+, 95.23), 292.00(19.84), 290.94(61.12), 250.92(29.51), 248.93(M^+-CO_2, 100.00), 248.00(15.08), 246.93(64.54), 170.01(54.20), 168.02(65.93), 143.00(15.17), 141.05(21.36), 97.22(15.29), 88.12(16.83), 61.15(14.95).

Synthesis of 5-iodo-2,1-benzisoxazole-3(1*H*)-one (2e, CAS: 773851-07-3): According to General procedure 2 the mixture of 13.4 mg (0.046 mmol) of 2-azido-5-iodobenzoic acid and 25.8 mg (0.46 mmol) of potassium hydroxide in 10 mL of ethanol was irradiated for 1.5 h. The reaction mixture was added to 100 mL of water containing 0.1 mL phosphoric acid and the mixture was extracted with benzene. The benzisoxazole-containing organic phase was eluted through silica gel using benzene. The benzisoxazole-containing solution was evaporated to dryness in vacuo affording 5-iodo-2,1-benzisoxazole-3(1*H*)-one with 51% yield as white crystals. m/z (rel, intensity) 261.90(11.16), 260.92(M^+, 100.00), 260.22(11.73), 259.34(8.10), 235.00(5.59), 216.96(M^+-CO_2, 34.90), 216.01(4.26), 134.21(6.31), 130.37(5.46), 129.09(4.35), 126.99(6.76), 90.08(26.07), 78.16(8.82), 77.07(7.23), 63.15(9.58).

Synthesis of 5-(triphenylmethyl)-2,1-benzisoxazole-3(1*H*)-one (2f, new compound): According to General procedure 2 the mixture of 15 mg (0.037 mmol) of 2-azido-5-(triphenylmethyl)benzoic acid and 3.8 mg (0.037 mmol) of sodium acetate

in 10 mL ethanol was irradiated for 1 h. The reaction mixture was evaporated to dryness, the residue dissolved in chloroform and eluted through silica gel. The benzisoxazole-containing solution was concentrated in vacuo affording crystals which were recrystallized from hexane to give of 5-(triphenylmethyl)-2,1-benzisoxazole-3(1*H*)-one with 39% yield as yellow crystals. ^{1}H NMR (400 MHz, $CDCl_3$) δ 7.31, 7.29, 7.27, 7.26, 7.26, 7.23, 7.22, 7.21, 7.19, 7.18, 7.16. ^{13}C NMR (101 MHz, $CDCl_3$) δ 166.8, 158.8, 153.9, 147.8, 146.3, 146.0, 145.8, 145.7, 145.6, 141.1, 138.5, 137.4, 135.2, 134.9, 131.0, 131.0, 130.9, 128.2, 128.1, 128.1, 128.0, 127.9, 127.8, 118.8, 115.8, 109.5, 77.2. IR (suspension in nujol oil, cm^{-1}): 1733 (ν C=O); 1596 (ν C=C); 1492 (ν N=O); m/z (rel, intensity) 380.09(M^++3, 13.00), 379.11(M^++2, 33.25), 303.12(21.96), 302.11(100.00), 301.24(15.41), 300.20(15.90), 284.11(24.73), 243.13(24.48), 239.11(17.76), 228.11(17.94), 227.17(10.68), 165.09(36.69)

Synthesis of 2-oxo-3*H*-azepine-3-carboxylic acid (3a, CAS: 134050-77-4): According to General procedure 2 the mixture of 350 mg (2.2 mmol) of 2-azidobenzoic acid in 12 mL of acetonitrile and 30 mL of water was irradiated for 24 h. The reaction mixture was evaporated to dryness and 2-oxo-3-carboxy-3*H*-azepin was isolated by preparative column chromatography on silica gel with a mixture of acetone/ethanol (1:1, v/v) with 50% yield as pale yellow crystals. ^{1}H NMR (400 MHz, $CDCl_3$) δ 8.35 (s, 1H), 6.31 – 6.17 (m, 2H), 5.93 (ddd, *J* = 14.1, 9.2, 5.5 Hz, 2H), 3.53 (dd, *J* = 5.8, 1.6 Hz, 1H). ^{13}C NMR (101 MHz, $CDCl_3$) δ 168.9, 164.6, 126.8, 125.5, 121.3, 114.8, 52.9, 52.8; IR (suspension in nujol oil, cm^{-1}): 3198 $см^{-1}$ ($ν_S$ NH); 1714 $см^{-1}$ ($ν_S$ C=O carboxyl group); 1658 $см^{-1}$ ($ν_S$ C=O amide) [7].

Synthesis of 5-bromo-2-oxo-3H-azepine-3-carboxylic acid (3c, new compound): According to General procedure 2 the mixture of 15.5 mg (0.064 mmol) of 2-azido-5-

bromobenzoic acid in 10 mL of 1,4-dioxane/water 1:10 was irradiated for 1.5 h. The reaction mixture was extracted with benzene to remove byproducts from the aqueous solution. The aqueous azepine-containing solution was evaporated to dryness in vacuo and the residue was dissolved in ethanol and eluted through silica gel using ethanol as the eluent. The azepine-containing solution was evaporated to dryness in vacuo affording 5-bromo-2-oxo-3*H*-azepine-3-carboxylic acid with 50% yield as pale yellow crystals. ^{1}H NMR (400 MHz, d6-DMSO) δ 10.35 (s, 1H), 6.30 (s, 1H), 6.00, 5.99 (d, J = 5.0 Hz, 1H), 5.90, 5.88 (d, J = 8.7 Hz, 1H), 3.51 (d, J = 6.6 Hz, 1H). ^{13}C NMR (101 MHz, d6-DMSO) δ 168.3, 163.4, 128.8, 120.5, 118.8, 114.0, 52.8, 39.5. IR (suspension in nujol oil, cm^{-1}): 3256 см$^{-1}$ (ν_s NH); 1725 см$^{-1}$ (ν_s C=O carboxyl group); 1688 см$^{-1}$ (ν_s C=O amide); m/z (rel, intensity) 188.97(M^++2-CO_2, 88.01), 188.08(19.87), 186.96(M^+-CO_2, 100.00), 186.13(13.19), 160.99(13.05), 159.98(25.88), 159.01(16.48), 157.98(24.72), 149.04(12.70), 144.97(7.98), 108.08(21.61), 103.08(9.86), 80.10(43.78), 79.09(10.40), 61.11(11.25).

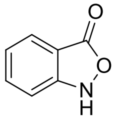

2a, ^1H NMR, $CDCl_3$

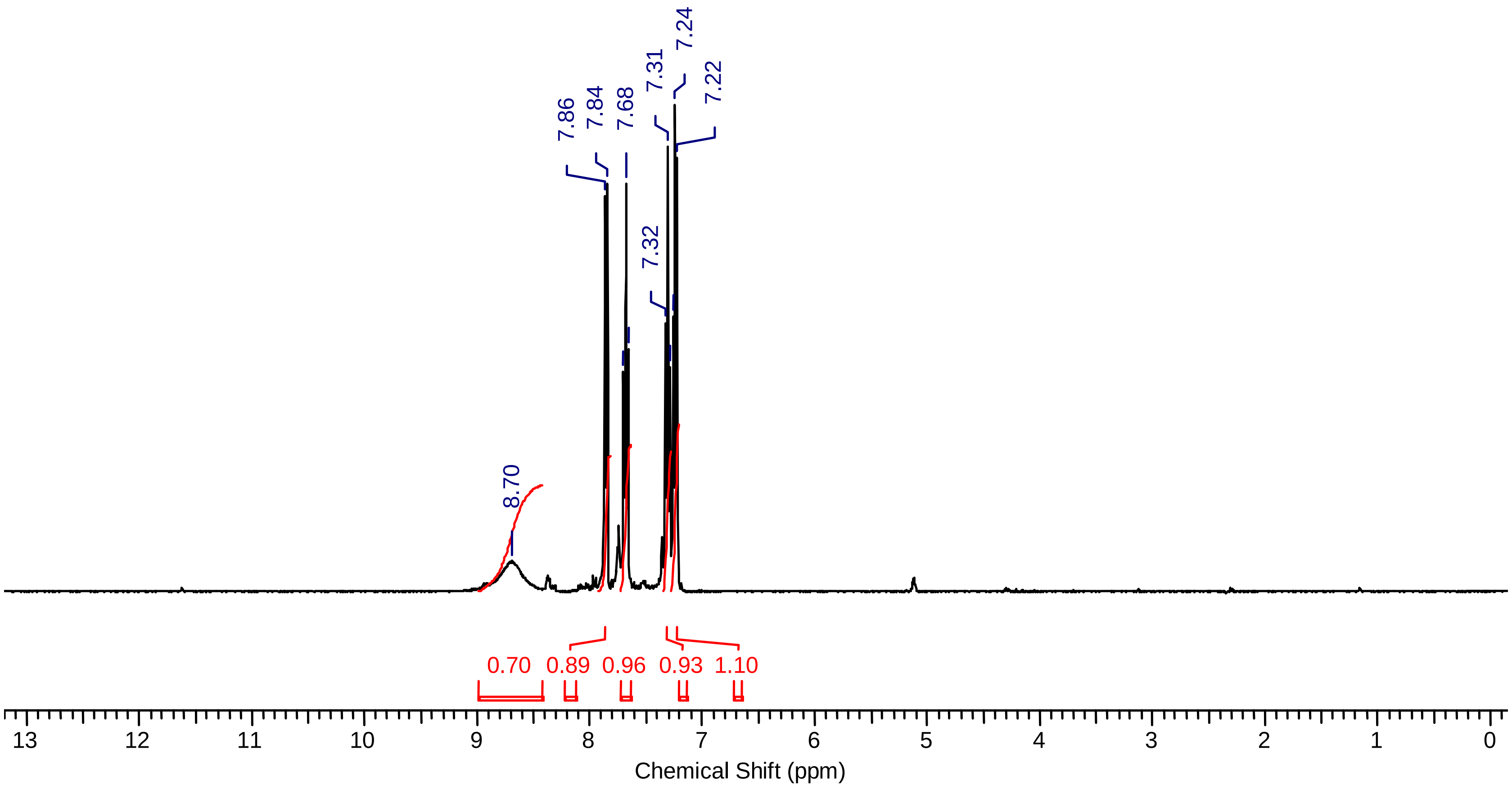

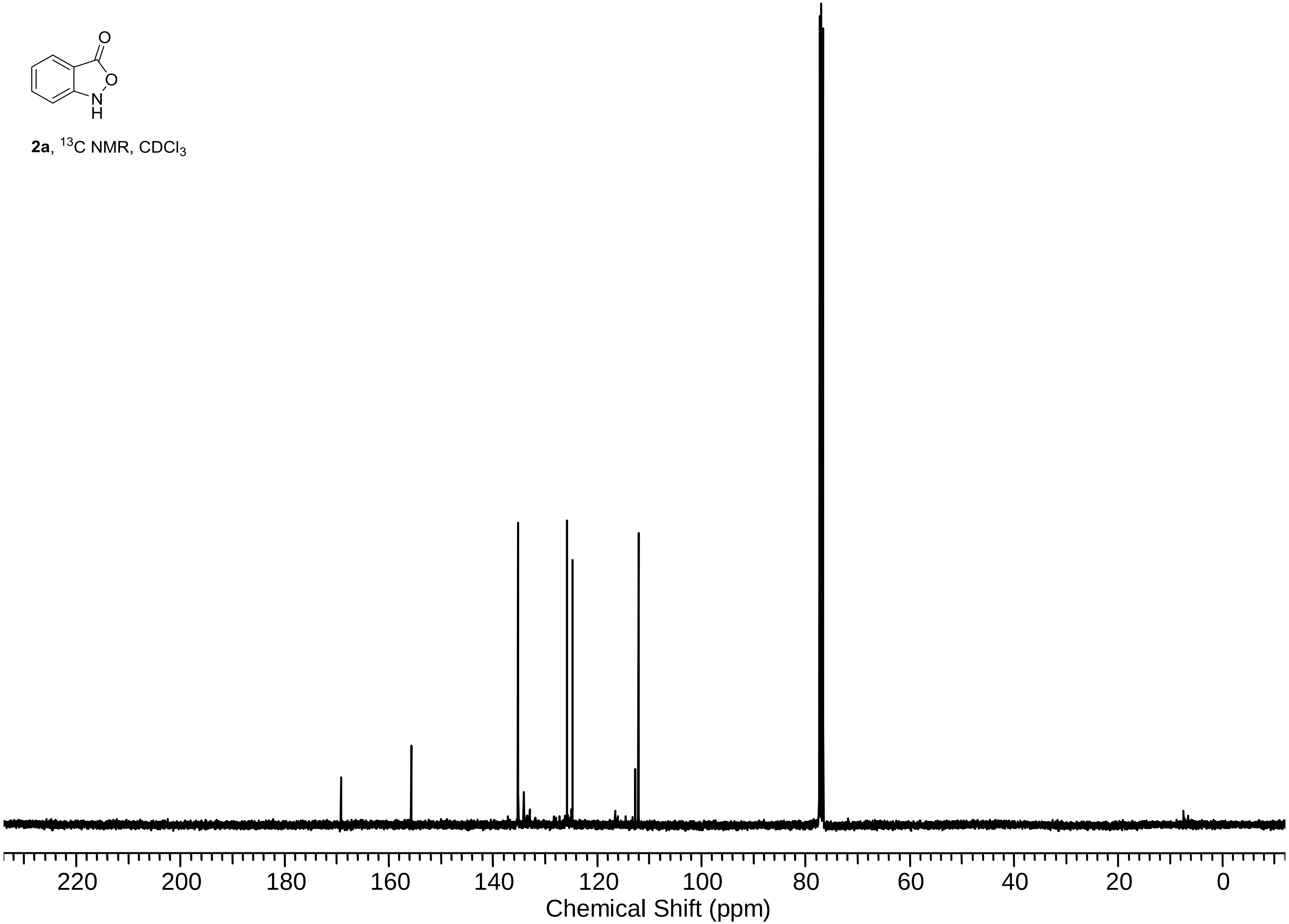

2a, ^{13}C NMR, $CDCl_3$

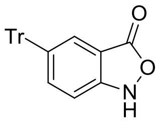

2f, ^{1}H NMR, CDCl3

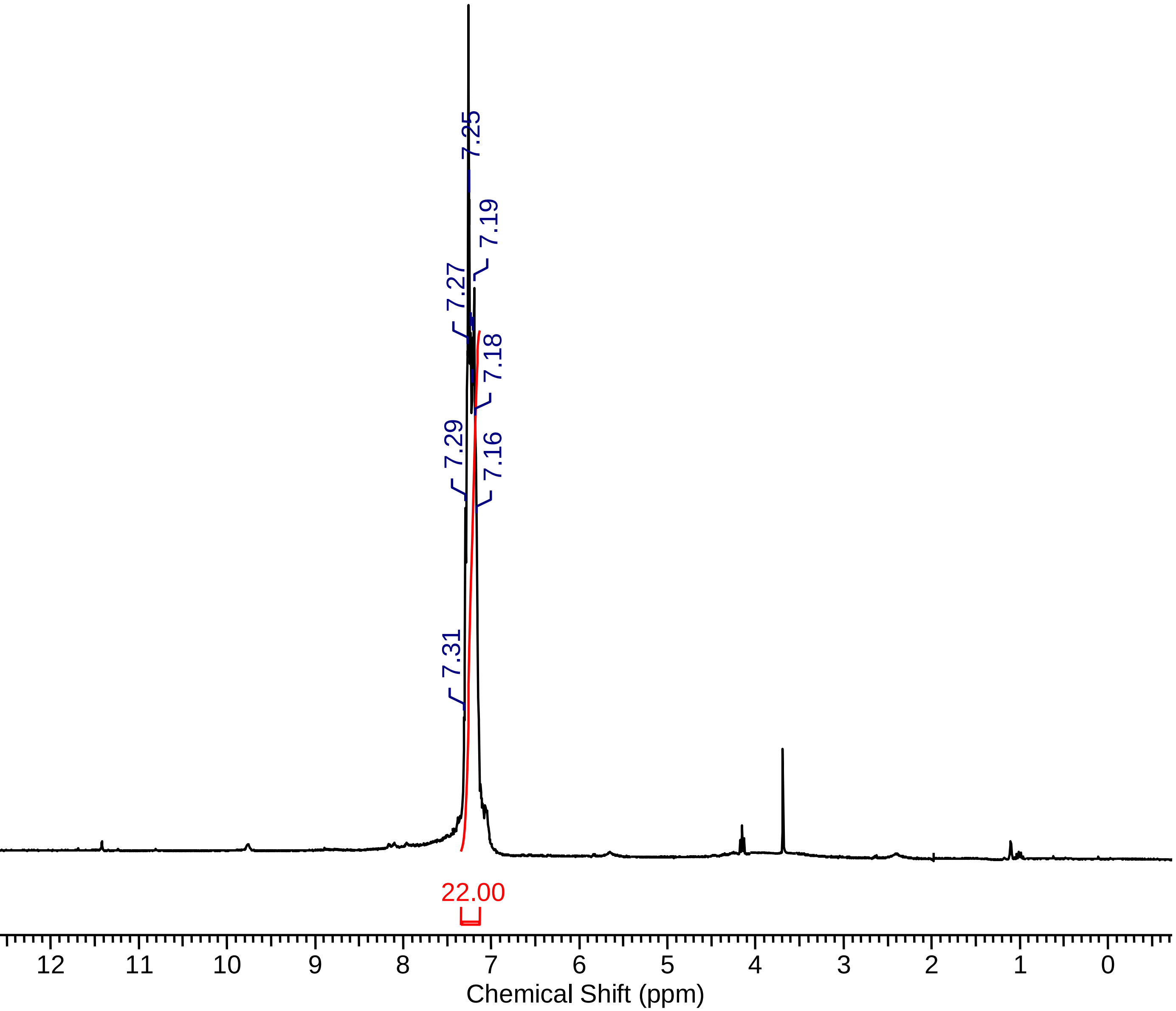

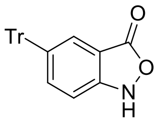

2f, ^{13}C NMR, CDCl3

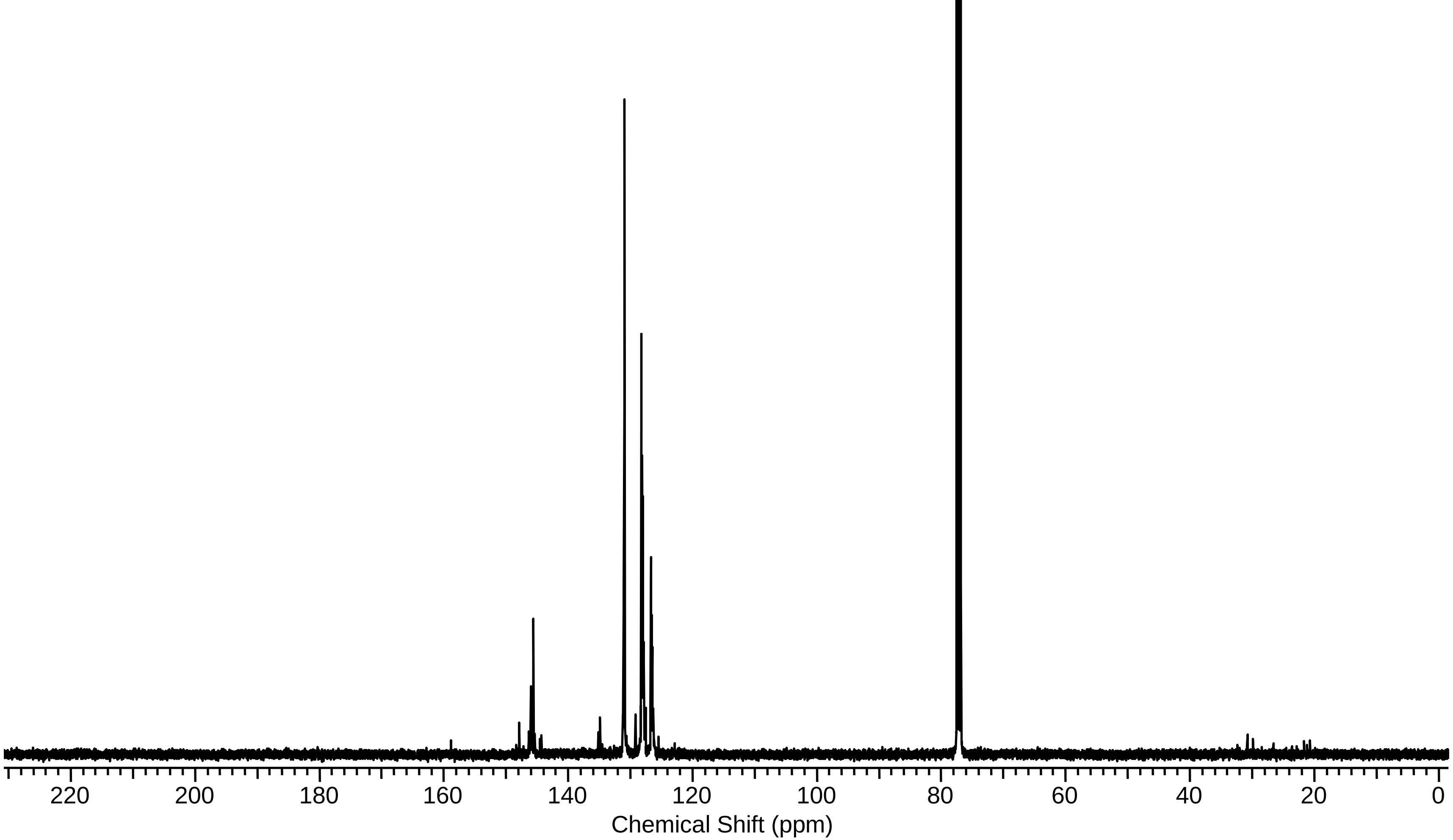

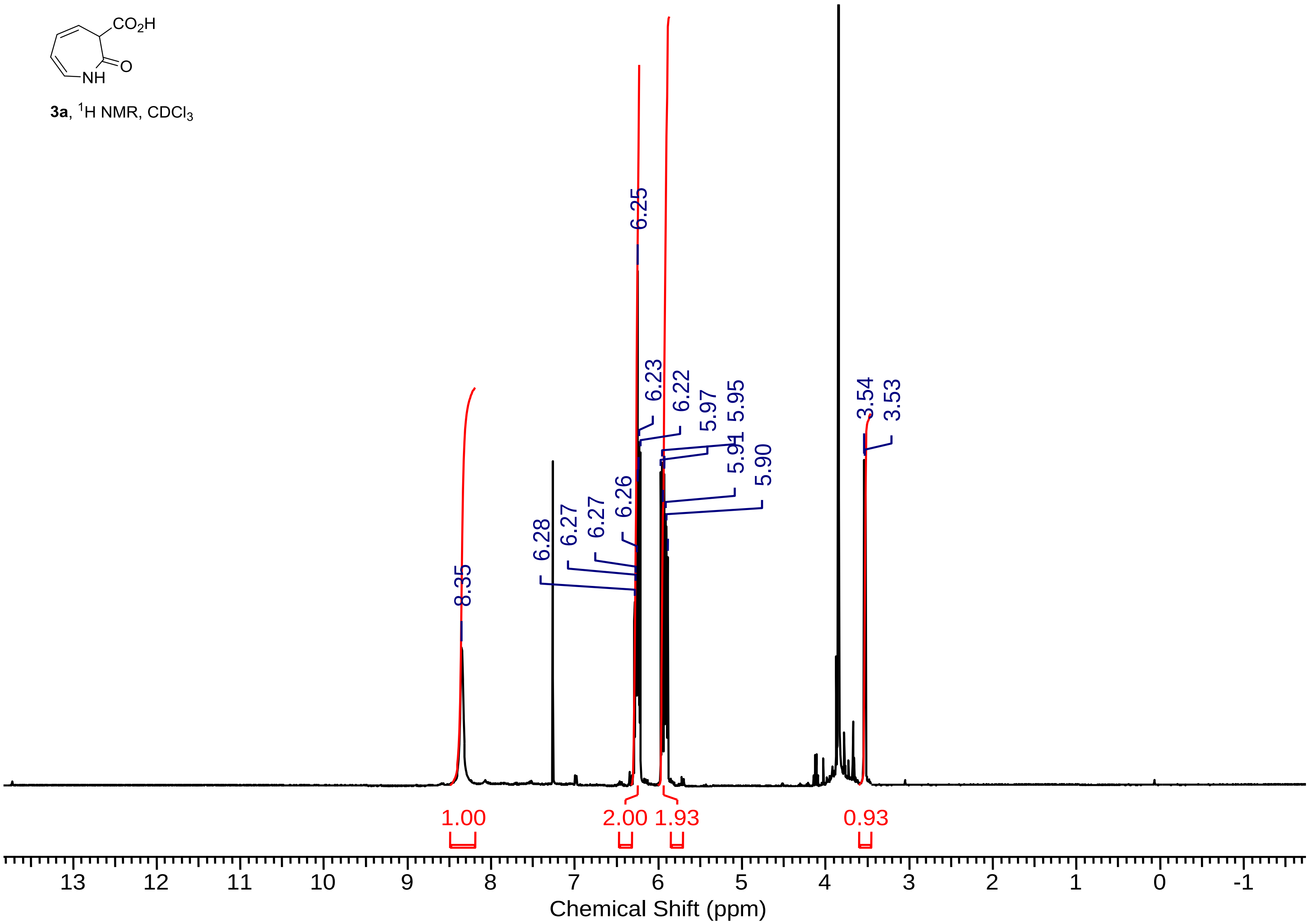

CO2H
NH
O
3a, 1H NMR, CDCl3
8.35
6.28
6.27
6.27
6.26
6.25
6.23
6.22
5.97
5.95
5.91
5.90
3.54
3.53
1.00
2.00
1.93
0.93
13
12
11
10
9
8
7
6
5
4
3
2
1
0
-1
Chemical Shift (ppm)

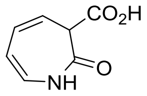

3a, ^{13}C NMR, $CDCl_3$

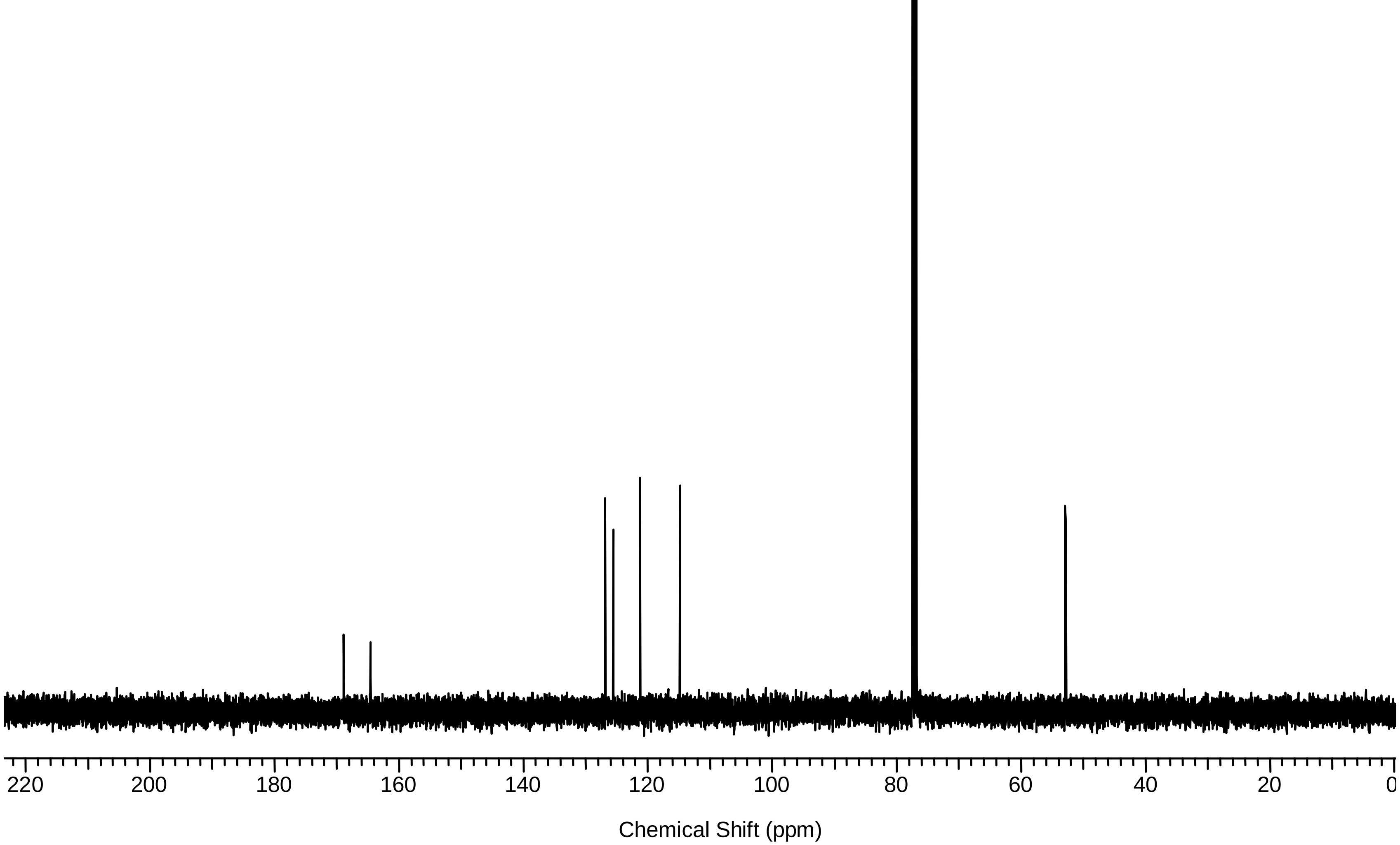

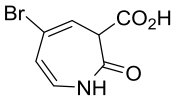

3c, ^{1}H NMR, d_6-DMSO

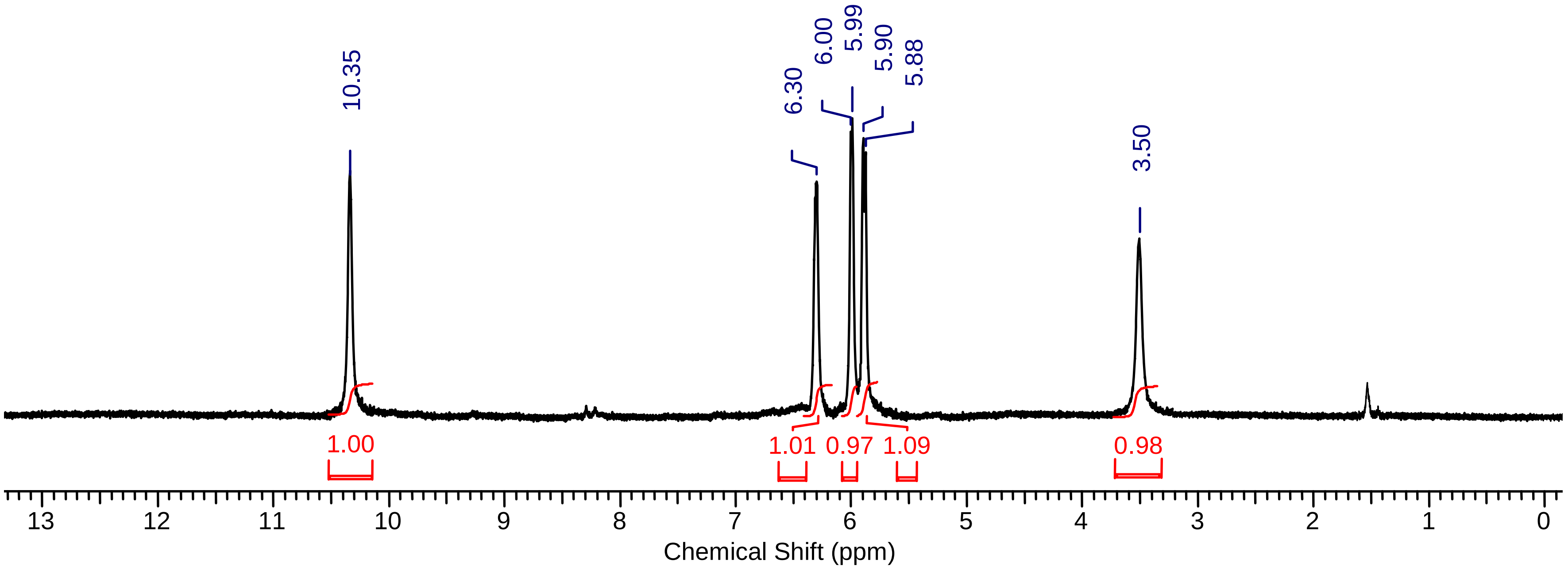

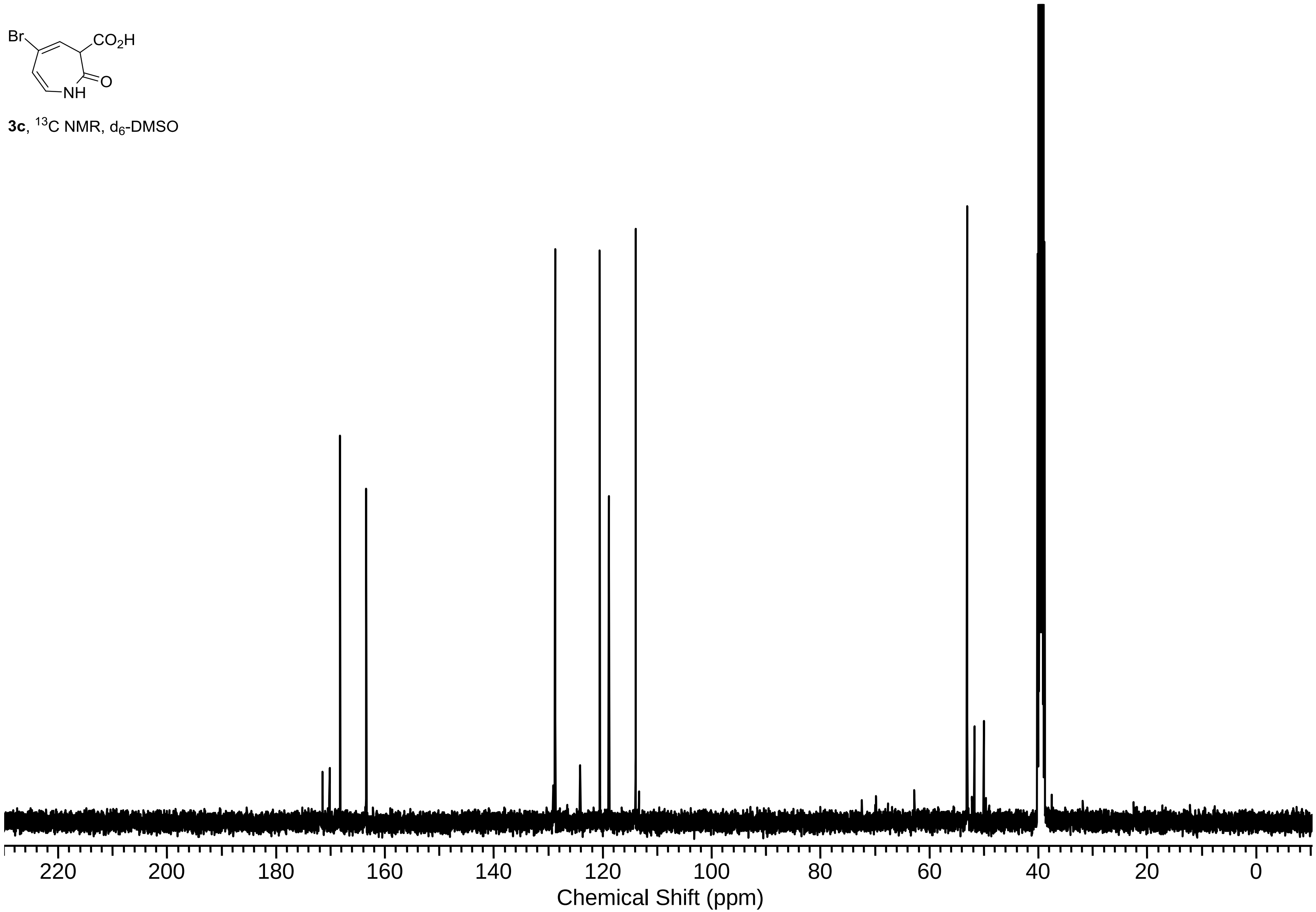
Br
CO2H
O
NH
3c, 13C NMR, d6-DMSO
220
200
180
160
140
120
100
80
60
40
20
0
Chemical Shift (ppm)

References

S1. Armarego, W. L. F.; Chai, C. Purification of laboratory chemicals; Butterworth-Heinemann, **2003**.

S2. Atkinson, E. R.; Mitton, P. B. *J. Am. Chem. Soc.* **1947**, 69 (12), 3142.

S3. Rajveer, Ch.; Kumaraswamy, D.; Sudharshini, S.; Rathinaraj, B.S. *Int. J. Pharm. Bio Sci.* **2010**, 1(3), 1 - 10.

S4. Yamada, K.; Fushimi, N. Method for producing 2-amino-5-iodobenzoic acid. Patent WO 2006016510 A1, May 27, **2008**.

S5. Benkeser, R. A.; Gosnell, R. B. *J. Am. Chem. Soc.* **1956**, 78 (19), 4914–4916.

S6. Budruev, A. V; Karyakina, L. N.; Levina, O. P.; Oleinik, A. V. *Russ. J. Coord. Chem.* **2005**, 31 (3), 181–184.

S7. Sinjagina, D. Y.; Budruev, A. V. *High Energy Chem.* **2013**, 47 (4), 162–165.
